# GSTT1 is upregulated by oxidative stress through p38-MK2 signaling pathway in human granulosa cells: possible association with mitochondrial activity

**DOI:** 10.18632/aging.100418

**Published:** 2011-12-28

**Authors:** Megumu Ito, Misa Imai, Miho Muraki, Kenji Miyado, Junwen Qin, Shigeru Kyuwa, Yasuhiro Yoshikawa, Yoshihiko Hosoi, Hidekazu Saito, Yuji Takahashi

**Affiliations:** ^1^ Division of Reproductive Medicine, Department of Perinatal Medicine and Maternal Care, National Center for Child Health and Development, Tokyo 157-8535, Japan; ^2^ Department of Biochemistry, Tufts University School of Medicine, Boston, MA 02111, USA; ^3^ Department of Biomedical Science, Graduate School of Agricultural and Life Sciences, The University of Tokyo, Tokyo 113-8657, Japan; ^4^ Department of Reproductive Biology, National Center for Child Health and Development, Tokyo 157-8535, Japan; ^5^ Institute of Reproductive Immunology and Key Laboratory for Regenerative Medicine, Ministry of Education, Jinan University, Guangzhou 510632, China; ^6^ Division of Biological Science, Graduate School of Biology-Oriented Science and Technology, Kinki University, Wakayama, 649-6493 Japan

**Keywords:** GSTT1, p38 MAPK, MK2, granulosa cell, aging, mitochondria

## Abstract

We previously reported that GSTT1 was upregulated in human granulosa cells during aging and that activation and localization of p38 MAPK was changed in parallel. Although oxidative stress is responsible for these changes, the age-associated expression of GSTT1 regulated by MAPKs and the role of GSTT1 in aged granulosa cells remain unclear. Therefore, we examined the relationship between the expression of GSTT1 and MAPK signaling pathways using human granulosa-like KGN cells stimulated with H_2_O_2_ in the presence or absence of various MAPK inhibitors. Interestingly, H_2_O_2_-induced GSTT1 was only inhibited by a p38 inhibitor. An inhibitor of MK2, a downstream regulator of p38, also diminished H_2_O_2_-induced GSTT1 upregulation. Notably, both p38 and MK2 were significantly inactivated in cells carrying an shRNA construct of GSTT1 (ΔGSTT1 cells), suggesting that the p38-MK2 pathway is essential for age-associated upregulation of GSTT1. The relevance of GSTT1 in mitochondrial activity was then determined. ΔGSTT1 cells displayed enhanced polarization of mitochondrial membrane potential without increasing the apoptosis, suggesting that the age-associated upregulation of GSTT1 may influence the mitochondrial activity of granulosa cells.

Collectively, it appears that the age-associated expression of GSTT1 is induced through the p38 signaling pathway and GSTT1 influences homeostatic activities in granulosa cells.

## INTRODUCTION

Glutathione S-transferases (GSTs) are well known for removing environmental pollutants and endogenous toxic compounds as part of the phase II detoxification process through glutathionylation of diverse electrophilic substrates. This self-defense system is highly conserved among all organisms including prokaryotes and eukaryotes [1]. Because of their well-known characteristics, the polymorphism of GSTs causing point or null mutations are often associated with certain diseases [2, 3]. There are 8 subclasses of GSTs in mammals [4], and some of these have been shown to act as antioxidants against reactive oxygen species (ROS). Overexpression of GSTA4 has been shown to protect cells from 4-hydroxynonenal (4-HNE)-induced apoptosis by inhibition of JNK signaling [5]; GSTP has also been associated with JNK and protects cells from death signals or oxidative stress [6]. Therefore, it is plausible that GSTs have a strong link to aging and are assured longevity [7]. This hypothesis has been proven in *Caenorhabditis elegans* in which CeGSTP2-2 belonging to the pi-class of GSTs was reported to conjugate 4-HNE and its overexpression was shown to elongate lifespan [8, 9]. In contrast, genetic disruption of GSTA4 in mice showed unexpected elongation of lifespan, probably due for compensation of the GSTA4 loss by other NRF2-dependent antioxidants [10].

The expression level of GSTs is decreased in various tissues and organs during aging [11], indicating that the cells have less protection against a number of toxins and oxidative stress at this time. However, GSTT1 is highly upregulated in aged human granulosa cells [12], although its relevance in reproductive aging remains to be elucidated. GSTT1 is thought to be the most ancient of GST classes and it possesses unique bilateral features [13]. It acts as a scavenger toward electrophiles of various toxins and protects cells and tissues as well as other GST classes. Susceptibility to certain cancers has been proposed to occur in conjunction with the GSTT1–null genotype, [14]. In contrast, GSTT1 produces formaldehyde hazardous for DNA from several halogenated compounds, such as dichloromethane, during its metabolism [15]. Indeed, endogenous formaldehyde levels have been reported to be elevated during aging [16]. GSTT1 has also been shown to induce significant decrease in cell viability in aortic endothelial cells in conjunction with oxidative stress [17]. Collectively, these results suggest that GSTT1 as a candidate molecule associated with aging, regardless of whether this molecule is useful or harmful for living organisms.

The p38 MAPK signaling pathway has been involved in a number of important biological activities, such as proliferation, inflammation, cell death, and aging [18]. The activation of p38 is dependent not only on stimuli but also on cell types. In reproductive cells, it plays a pivotal role in oocyte maturation [19-22] and steroidogenesis [23, 24]. On the other hand, p38, similar to JNK, is known to function as a stress transducer, and is highly activated in aged cells and tissues [25-27]. p38 is activated in klotho knockout mice showing a premature aging phenotype, whereas it is down-regulated in klotho-overexpressing model [28]. In addition, a p38 inhibitor prevented death of fibroblasts from Werner syndrome *in vitro* [29, 30]. Therefore, p38 is involved in ROS-induced cellular damage during aging. Interestingly, p38 is activated in the cytoplasm of aged granulosa cells, whereas it is phosphorylated in the nucleus of younger cells [31]. Since p38 has been shown to translocate between the nucleus and cytoplasm in response to various stimuli [32, 33], the downstream transporters of p38 including MK2, MK5 and TAB-1 [32, 34], must be involved in age-associated change in the subcellular localization of p38.

Some GSTs have been shown to be upregulated through the MAPK pathways as self-defense responses to toxins and growth factors [35, 36]. However, MAPKs that regulate GSTT1 expression and functions have not yet been reported. Furthermore, there is no clear under-standing of the roles of GSTT1 during aging. Therefore, we attempted to determine the direct implications of the MAPK pathways in the expression of GSTT1. We also studied the involvement of GSTT1 in mitochondrial activity.

## RESULTS

### Regulation of H_2_O_2_–induced GSTT1 by p38 MAPK

**Figure 1:**
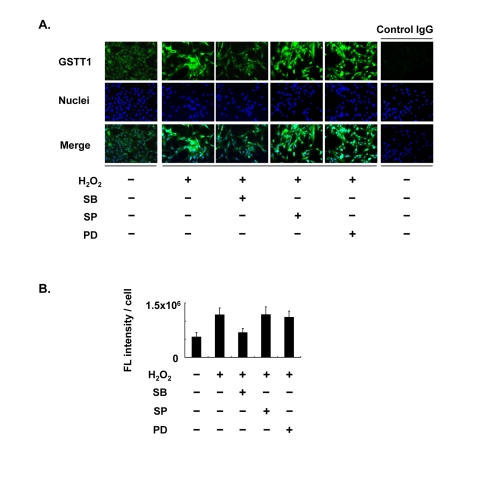
Effects of MAPK inhibitors on the expression of GSTT1 in KGN cells stimulated with H2O2. Cells were treated with H_2_O_2_ at 200 μM in the presence or absence of SB203580, SP600125 or PD98059 at 10 μM for 24 h and then subjected to the immunofluorescence analysis (**A**). The primary antibody against GSTT1 was probed with anti-rabbit IgG-Alexa488 (Green). Cells were counterstained with Hoechst 33342 at 10 μM (Blue). Magnification: ×200. A bar graph represent the mean fluorescence intensity per cell ± SEM (**B, C**). One-way ANOVA: (**B**) P < 0.05; (**C**) P < 0.01.

In our previous studies, we observed age-associated changes in GSTT1 expression in granulosa cells [12], as well as changes in the subcellular localization of p38 [31]. Although H_2_O_2_ is able to induce these changes *in vitro*, it remains uncertain whether GSTT1 induction by H_2_O_2_ is controlled through the p38 MAPK signaling pathway. Therefore, we examined the expression of GSTT1 induced by H_2_O_2_ in the presence or absence of distinct MAPK inhibitors. The immunofluorescence study revealed that GSTT1 was highly upregulated in response to H_2_O_2_, as has been reported previously (Figure 1). In the cells stimulated with H_2_O_2_, only SB203580 prevented the upregulation of GSTT1 (Figure 1B: ANOVA, P < 0.05). These results strongly suggest that the p38 signaling pathway specifically regulates H_2_O_2_-induced GSTT1.

### GSTT1 is involved in the p38-MK2 signal cascade under oxidative stress

**Figure 2:**
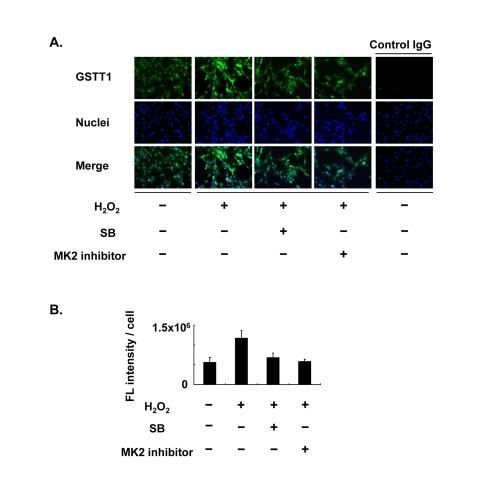
Effects of the MK2 inhibitor on the expression of GSTT1 in KGN cells stimulated with H_2_O_2_ Cells were treated with H_2_O_2_ at 200 μM with or without SB203580 at 10 μM or CMPD1 at 330 nM for 24 h and subjected to immunofluorescence analysis (**A**). The primary antibody against GSTT1 was probed with anti-rabbit IgG-Alexa488 (Green). Cells were counterstained with Hoechst 33342 at 10 μM (Blue). Magnification: ×200. A bar graph represent the mean fluorescence intensity per cell ± SEM (**B**, **C**). One-way ANOVA: (**B**) P < 0.05; (C) P < 0.01.

Since one of the p38 downstream targets, MK2, has been shown to be responsible for both translocation and stress signaling of p38, MK2 may play a role in the regulation of GSTT1 in granulosa cells. KGN cells were therefore pretreated with CMPD1, an MK2 inhibitor, prior to stimulation with H_2_O_2_ (Figure 2). As expected, CMPD1 blocked GSTT1 induction by H_2_O_2_ very efficiently, which is very similar to the effects of SB203580 (Figure 2B).

**Figure 3:**
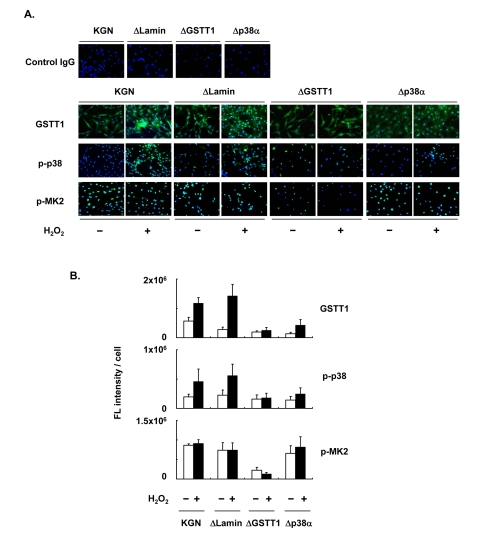
Depletion of GSTT1 inactivates the p38–MK2 signaling pathway. KGN cells (wild–type, ΔLamin ΔGSTT1 or Δp38α cells) were treated with or without H_2_O_2_ at 200 μM for 24 h before investigation of the expression of GSTT1 and activation of p38 and MK2 by immunofluorescence analysis (**A**). The primary antibodies against GSTT1, phosphorylated p38 and phosphorylated MK2 were probed with anti-rabbit IgG–Alexa488 (Green). Cells were counterstained with Hoechst 33342 at 10 μM (Blue). Magnification: ×200. Bar graphs represent the mean fluorescence intensity per cell ± SEM (**B**). One–way ANOVA: (**B**, GSTT1) P < 0.001, (**B**, p-p38) P < 0.05, (**B**, p-MK2) P < 0.001.

For further analysis of the stress-induced regulation of GSTT1 in granulosa cells, KGN cells stably carrying a knockdown construct of either GSTT1 or p38α were established (ΔGSTT1 and Δp38α cells), and the expression of GSTT1, as well as the phosphorylation of p38 and MK2 was examined for each cell line (Figure 3). H_2_O_2_ induced GSTT1 expression and phosphorylation of p38 very efficiently in wild-type and ΔLamin (cells carrying the control vector) cells, whereas both were severely suppressed in ΔGSTT1 and Δp38α cells (Figure 3B). More interestingly, the phosphorylation of MK2 was significantly impaired only in ΔGSTT1 cells and was not even activated by the addition of H_2_O_2_, suggesting that GSTT1 is highly correlated with MK2 activity.

**Figure 4:**
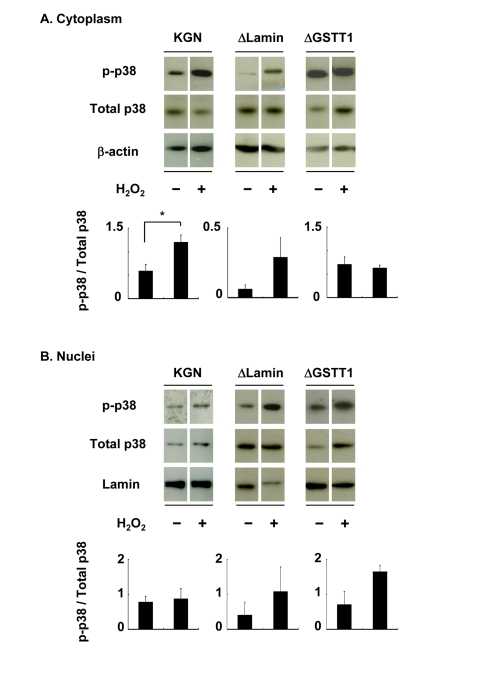
Depletion of GSTT1 prevents the cytoplasmic activation of p38. Cells stimulated with or without H_2_O_2_ were subjected to fractionation of cytosolic and nuclear proteins. The activity of p38 in each fraction was then analyzed by immunoblotting (**A**: cytoplasm, **B**: nuclei). Fifteen micrograms of total protein were used for each lane. Bar graphs represent the mean band intensity ± SEM. (**A**) Student's *t*–test: P < 0.05.

Supporting the above results, subcellular phosphorylation of p38 in ΔGSTT1 cells differed from that in wild-type and ΔLamin cells (Figure 4). While p38 showed greater phosphorylation in the cytoplasm of wild-type and ΔLamin cells after treatment with H_2_O_2_, it was unchanged in ΔGSTT1 cells (Figure 4A). In contrast, the nuclear p38 in all cell types was not significantly different before and after treatment with H_2_O_2_, although it appeared to be increased in ΔLamin and ΔGSTT1 cells compared with wild-type cells (Figure 4B). These results strongly suggest that GSTT1 is involved in the p38-MK2 signaling pathway.

### GSTT1 is involved in mitochondrial activity

**Figure 5:**
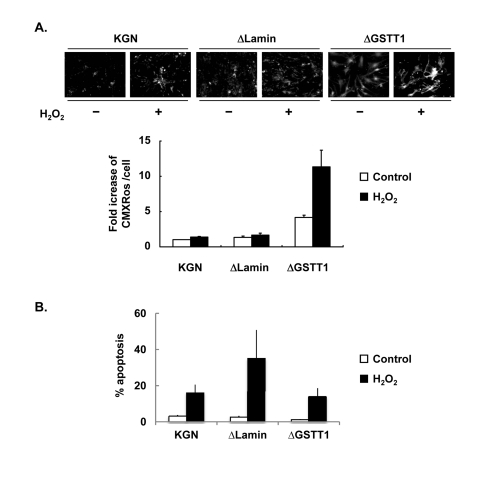
Depletion of GSTT1 enhances mitochondrial activity. (**A**) Wild-type, ΔLamin or ΔGSTT1 cells were stimulated with H_2_O_2_, and the mitochondrial membrane potential of each cell type was observed by staining with Mitotracker CMXRos at 100 nM. A bar graph represent the fold increase in mean fluorescence intensity ± SEM. One–way ANOVA: P < 0.001. (**B**) Frequency of apoptosis before and after treatment with H_2_O_2_ was measured by TUNEL assay. The number of apoptotic cells was counted and divided by the total number of cells per field. A bar graph represent the frequency of apoptosis ± SEM.

Because the expression of GSTT1 induced by oxidative stress is mediated through the p38 signaling pathway, GSTT1 may influence the mitochondrial activity. Therefore we examined the ΔΨm of ΔGSTT1 cells using Mitotracker CMXRos. As shown in Figure 5A, suppression of the GSTT1 expression led to a marked increase in the fluorescence intensity of CMXRos under normal conditions. In addition, the mitochondrial hyperpolarity was much more intense in ΔGSTT1 cells compared with wild-type and ΔLamin cells after treatment with H_2_O_2_.

Since the mitochondrial membrane potential is closely related to cellular viability, the apoptosis of these cells after treatment with 200 μM H_2_O_2_ was determined by TUNEL assay. As shown in Figure 5B, the viability of ΔGSTT1 cells before and after H_2_O_2_ treatment was comparable to that of wild-type KGN cells, indicating that the hyperpolarization of ΔΨm observed in ΔGSTT1 cells is not a sign of senescence.

## DISCUSSION

Expression of GSTs is directly or indirectly regulated through MAPK pathways. In hepatocytes, GSTM1 and M2 are upregulated by geniposide, an irdoid glycoside, through the ERK signal pathway [36], and an ERK inhibitor was shown to enhance upregulation of GSTA1 by sulforaphane in CaCo-2 cells [37]. In Hep3B cells, GSTA1 has been shown to be induced by alcohol through both ERK and p38 pathways [38]. Similarly, EGF-dependent induction of GSTA4 is mediated by the ERK and p38 pathways [35]. Therefore, the induction of each GST class through distinct MAPK pathways is likely to be cell type- and stimulus-specific. In this study, GSTT1 was shown to be upregulated in human granulosa cells by oxidative stress only through the p38 signal pathway, suggesting that the interplay between p38 and GSTT1 may be involved in aging of granulosa cells.

MK2 is known to be a direct substrate of p38, and determines the subcellular localization of p38 [39]. Although it remains unclear whether the age-associated subcellular localization of p38 in granulosa cells is caused by the action of MK2, the data shown in Figure 2 indicate not only that oxidative stress-induced GSTT1 is mediated specifically through the p38-MK2 pathway, but also that aged-related functional modifications in granulosa cells may be dependent on the action of MK2. MK2 is regarded as a versatile molecule; it functions in proinflammatory cytokine stabilization [39] and also modulates proteolysis through direct interactions with Hsp27 [40]. These functions are closely associated with the activity and localization of p38. Endogenous p38 is located in both the cytoplasm and nucleus of the resting cells, and cytoplasmic p38 translocates into the nucleus in response to various stimuli [32]. Activated p38 then phosphorylates nuclear MK2 and forms a complex whereby the MK2 nuclear export signal is unmasked, resulting in its rapid export from the nucleus [33, 41]. In contrast, p38 has also been reported to be down-regulated in MK2-deficient tissues [39, 42]. Regarding the amount of total MK2, it was decreased markedly in ΔGSTT1 cells in our preliminary experiments (data not shown). These results may demonstrate that GSTT1 is one of the target molecules for MK2 controlled by the p38 signaling pathway and may be involved in cytokine production. Further study is required.

Fluctuation in ΔΨm is important for mitochondrial activity. ΔΨm is generally accepted to be decreased by the opening of pores in the mitochondrial inner membrane in response to oxidative stress, resulting in apoptotic and necrotic cell death [43]. More precisely, increasing ΔΨm is observed only at the early onset of apoptosis [44], and triggers cytochrome c release into the cytosol, collapse of ΔΨm, activation of caspases, and DNA fragmentation [45]. On the contrary, neurons with more hyperpolarization are associated with increased glucose uptake, NADPH availability and increased viability [46]. Also, the following feedback mechanism has been suggested; glucose-induced mitochondrial hyperpolarization leads to a rise in Ca^2+^, and the increased Ca^2+^ in turn decreases ATP synthesis by mitochondrial depolarization, terminating the influx of Ca^2+^ [47]. Thus, ΔΨm functions as a signal transducer to determine subsequent cellular activities.

Although the mechanism underlying the significant increase in ΔΨm in ΔGSTT1 cells remains to be determined, the lowered activity of p38 before and after H_2_O_2_ treatment in ΔGSTT1 cells may be related to hyperpolarization. In fact, H_2_O_2_ has been shown to induce activation of p38 and depolarization of ΔΨm in intestinal epithelial cells [48]. These changes were attenuated by pretreatment of cells with SB203580. Similar effects of SB203580 were observed in hepatocytes treated with arachidonic acid, a model for alcohol-induced liver injury [49].

The remaining question relating to our results must be whether or not the increase in ΔΨm observed in the context of lowered GSTT1 is beneficial for granulosa cells. As shown in Figure 5, the hyperpolarization of ΔΨm in ΔGSTT1 cells was not associated with cellular apoptosis. Rather, the increased basal ΔΨm in ΔGSTT1 cells may be due to the enhanced activity of mitochondria, since mitochondria-related steroidogenic genes are upregulated under normal condition (data not shown). The expression of one such gene, steroidogenic acute regulatory protein, is closely related to mitochondrial activity since it is significantly decreased after exposure of cells to oxidative stress [50]. We also observed increased expression of cyclooxygenase 2 in H_2_O_2_-stimulated ΔGSTT1 cells in our preliminary experiments. Therefore, the observed mitochondrial hyperpolarization may enhance the susceptibility of mitochondria to various external stimuli.

In conclusion, this study demonstrates that oxidative stress-induced upregulation of GSTT1 is mediated through the p38-MK2 signaling pathway. The changes in signal transduction induced by oxidative stress, in turn, influences the mitochondrial activity. Collectively, these results suggest that GSTT1 is associated with reproductive aging through its effects on the p38–MK2 signaling pathway.

## MATERIALS AND METHODS

### Reagents

Hydrogen peroxide (H_2_O_2_) was purchased from Wako Pure Chemical Co. Ltd (Tokyo, Japan). Hoechst 33342, protease inhibitor cocktail, phosphatase inhibitors 1 and 2, and a rabbit polyclonal antibody against phosphorylated p38 (Thr180/Tyr182) for the immunofluorescence analysis were purchased from Sigma-Aldrich Inc (Tokyo, Japan). SB203580 (p38 inhibitor) and rabbit polyclonal antibodies against GSTT1 and phosphorylated MK2 (Thr222) were purchased from Santa Cruz Biotechnology Inc (Santa Cruz, CA, USA). SP600125 (JNK inhibitor) was purchased from Enzo Life Sciences, Inc. (Farmingdale, NY, USA). PD98059 (ERK inhibitor) and CMPD1 (4-(2'-fluorobiphenyl-4-yl) - N -(4-hydroxyphenyl) - buty-ramide, MK2 inhibitor) were purchased from Merck Ltd. (Tokyo, Japan). A rabbit antibody against phosphorylated p38 (Thr180/Tyr182) for the immunoblot analysis was purchased from Novus Biologicals Inc. (Littleton, CO, USA). A mouse monoclonal antibody against β-actin, a control rabbit IgG, a control mouse IgG, and a donkey anti–mouse IgG conjugated with HRP were purchased from Chemicon International Co. Ltd. (Temecula, CA, USA). A goat anti–rabbit IgG conjugated with HRP was purchased from Thermo Fisher Scientific Inc. (Rockford, IL, USA). A rabbit polyclonal antibody against lamin was purchased from Abcam Inc. (Cambridge, MA, USA). A goat anti–rabbit IgG conjugated with Alexa Flour 488 and MitoTracker CMXRos were purchased from Molecular Probes, Inc. (Eugene, OR, USA).

### Cell culture and treatment

The human granulosa-like cell line, KGN, was maintained as described previously [12, 51]. Briefly, cells were cultured in DMEM/F12 containing 1 IU/ml penicillin, 1 μg/ml streptomycin, and 10% heat-inactivated FBS (Biowest Ltd., Nuaille, France, culture medium). Cells were seeded at 5 × 10^4^ cells / ml (200 ml / well) in 8-well culture slides (BD Japan Co. Ltd., Tokyo, Japan) for the subsequent immunofluorescence studies or in culture dishes (Nunc, Roskilde, Denmark) for immunoblot analysis. The medium was replaced by DMEM/F12 containing 0.1% BSA (serum-free medium) before stimulation. The cells were then treated with 200 μM H_2_O_2_ for 24 h with or without SB203580 (10 μM), SP600125 (10 μM), PD98059 (10 μM) or CMPD1 (330 nM). The final concentrations of inhibitors are determined according to the previous reports in which these inhibitors were used in various cell types [52-54]. After the treatment, they were either fixed or collected and stored as described previously [12, 31].

### shRNA design and transfection

To construct shRNA knockdown vectors, the target sequences of the desired genes were synthesized and obtained from Invitrogen Corp. (Carlsbad, CA, USA). GSTT1: Top CACCGCAGGAATGGCTTGCTTAAGACGAATCTTAAGCAAGCCATTCCTGC, Bottom AAAAGCAGG AATGGCTTGCTTAAGATTCGTCTTAAGCAAGCCATTCCTGC, p38α: Top CACCGCCGAGCTGTTGA CTGGAAGACGAATCTTCCAGTCAACAGCTCGGC, Bottom AAAAGCCGAGCTGTTGACTGAAGATTC GTCTTCCAGTCAACA GCTCGGC, Lamin: Top CACCGCTGGACTTCCAGAAGAACACGAATGTTCTTCTGGAAGTCCAG, Bottom AAAACTGGACTCC AGAAGAACATTCGTGTTCTTCTGGAAGTCCAGC, These oligonucleotides were annealed and cloned into a BLOCK–iT RNAi entry vector (pENTER/U6, Invitrogen Corp.) according to the manufacturer's instruction. After verification by sequencing of the DNA sequence inserted into the vector, the target sequences were transferred into the destination vector (pLenti6/BLOCK–iT-DEST) using the Gateway system (Invitrogen Corp.). Lentiviruses were then produced in 293FT cells using ViraPower Lentiviral Expression Systems, and KGN cells were subjected to lentiviral infection. Cells stably carrying each shRNA construct were maintained in culture medium containing blasticidin at 5 μg/ml. The efficiency of gene knock-down by shRNA was verified by either immunofluorescence or immunoblot analyses.

### Immunofluorescence

Immunofluorescence staining was performed as described previously [12]. Briefly, the cells were permeabilized and blocked with Block Ace (Snow Brand Milk Products Co. Ltd., Tokyo, Japan) and treated with the first antibody (10 μg/ml for p-p38 and p-MK2, 20 μg/ml for GSTT1) overnight at 4°C followed by treatment with the secondary antibody (1:200 dilution) for 2 h at room temperature. Hoechst 33342 at 10 μM was also included during treatment with the secondary antibody to enable detection of nuclei. Microphotographs were taken using an epifluorescence microscope equipped with a computational CCD camera (Olympus, Tokyo, Japan). For the image analysis, photographs were taken from five different areas in each sample using the MetaMorph program (Molecular Devices Corp. Tokyo, Japan), and the total fluorescence intensity was measured for each field. The number of cells per field was counted simultaneously by careful observation of Hoechst stained specimens. The normalized fluorescence intensity was then obtained by dividing the total fluorescence intensity by the number of cells in each field. Data were collected from three independent experiments and are shown as the mean fluorescence per cell ± SEM.

### Immunoblot analysis

KGN cells were passaged and cultured in a culture medium for 3 days (usually they reach to the sub-confluent status during the culture period) and then maintained for 6 h in a serum-free medium before treatment with H_2_O_2_ After treatment, the cells were washed twice with Tris-buffered saline (20 mM Tris, pH 7.5, 130 mM NaCl, TBS) containing protease inhibitor and phosphatase inhibitor cocktails and placed on ice as quickly as possible. They were then lysed by direct application of a lysis buffer to the dish.

The nuclear/cytosol fractionation kit (Bio Vision Technology Inc., New Minas, NS, Canada) was used to separate nuclear and cytoplasmic proteins, according to the manufacturer's protocol. After isolation of the proteins, the concentration of the samples was determined using a micro BCA assay kit (Thermo Fisher Scientific Inc.). Fifteen micrograms of sample per lane were electrophoresed on a 12% reducing SDS-polyacrylamide gel and transferred onto a PVDF membrane (Immobilon-P; Millipore, Tokyo, Japan). After blocking with 10% goat serum and 90% Block Ace at room temperature for 1 h, the membranes were treated with a primary antibody at 1:1000 dilution in TBST and subsequently with a secondary antibody at 1:10,000 dilution. SuperSignal West Femto Maximum Sensitivity Substrate (Thermo Fisher Scientific Inc.) was then used for visualization, and the signal was developed on an X-ray film (GE Healthcare, Piscataway, NJ, USA). The band intensity of phosphorylated p38 was measured using Adobe Photoshop Elements 2.0 software and the background was subtracted. It was then divided by the band intensity of total p38 for normalization. Data were collected from three independent experiments and are shown as the mean changes in band intensity ± SEM.

### Measurement of mitochondrial membrane potential (ΔΨm)

To assess the changes in ΔΨm, the cells were treated with or without 200 μM H_2_O_2_ for 24 h and labeled with the MitoTracker CMXRos probe at 100 nM for 30 min. They were washed twice with TBS and fixed with 4% formaldehyde for 1 h at room temperature. Fluorescence images were taken as described above and the fluorescence intensity of each image was measured with Adobe Photoshop Elements 2.0. It was then divided by the number of cells in each field. The fluorescence intensity per cell was defined as 1.0 for the wild-type KGN cells to obtain fold changes in fluorescence. Data were collected from three independent experiments and are shown as the mean fold increase in fluorescence ± SEM.

### Terminal deoxynucleotidyl transferase-mediated dUTP nick end-labeling (TUNEL) assay for apoptosis

Cell apoptosis was detected using TUNEL enzyme and labeling mix according to the manufacturer's instructions (Roche Diagnostics Japan Co. Ltd., Tokyo, Japan). In brief, cells were plated in 8-well culture slides for a couple of days and stimulated with 200 μM H_2_O_2_ for 24 h as described above. They were then washed with PBS three times and fixed with 2% paraformaldehyde in PBS for 1 h. Next, the cells were permeabilized and labeled with flurescein-dUTP. During the TUNEL labeling Hoechst 33342 at 10 μM was included. The number of flurescein-labeled cells and the total number of cells stained with Hoechst 33342 was counted simultaneously in each field of view under an epi-fluorescence microscope. The incidence of apoptosis was calculated by dividing the number of TUNEL-positive cells by the total number of cells per field. The experiments were repeated three times, and the data are shown as the mean percentage of apoptosis ± SEM.

### Statistical analysis

For all immunostaining experiments, statistical analysis was conducted by one-way ANOVA. For the immunoblot analysis, differences in the phosphorylation of p38 in cells treated with or without H_2_O_2_ were analyzed by Student's t-test or modified Student's t-test (Welch's correction) following an F test. For the analysis of apoptosis, a one-way ANOVA followed by Student's t-test was used. Differences were considered statistically significant if P < 0.05. All statistical analyses were performed using Microsoft Excel software.
